# Protective Properties of a Microstructure Composed of Barrier Nanostructured Organics and SiO*_x_* Layers Deposited on a Polymer Matrix

**DOI:** 10.3390/nano8090679

**Published:** 2018-08-31

**Authors:** Radek Prikryl, Pavel Otrisal, Vladimir Obsel, Lubomír Svorc, Radovan Karkalic, Jan Buk

**Affiliations:** 1 Institute of Materials Science, Faculty of Chemistry, Brno University of Technology, Purkynova 464/118, 612 00 Brno, Czech Republic; prikryl@fch.vut.cz; 2 Nuclear, Biological and Chemical Defence Institute of the University of Defence, Sidliste Vita Nejedleho, 68201 Vyškov, Czech Republic; 3 DEZA, Hochmanova 1, 628 01 Brno, Czech Republic; vobsel@seznam.cz; 4 Institute of Analytical Chemistry, Faculty of Chemical and Food Technology, Slovak University of Technology in Bratislava, Radlinskeho 9, 812 37 Bratislava, Slovakia; lubomir.svorc@stuba.sk; 5 Department of Military Chemical Engineering, Military Academy, GeneralaPavlaJurisicaSturma 33, 11000 Belgrade, Serbia; radovan.karkalic@va.mod.gov.rs; 6 P A R D A M, Ltd., Zizkova 2494, 413 01 Roudnicenad Labem, Czech Republic; jan.buk@pardam.cz

**Keywords:** barrier material, nanocoating of SiO*_x_*, polymeric matrix, plasma deposition, PVD, PA-PVD, PECVD, permeation, CERAMIS^®^, SorpTest

## Abstract

The SiO*_x_* barrier nanocoatings have been prepared on selected polymer matrices to increase their resistance against permeation of toxic substances. The aim has been to find out whether the method of vacuum plasma deposition of SiO*_x_* barrier nanocoatings on a polyethylene terephthalate (PET) foil used by Aluminium Company of Canada (ALCAN) company (ALCAN Packaging Kreuzlingen AG (SA/Ltd., Kreuzlingen, Switzerland) within the production of CERAMIS^®^ packaging materials with barrier properties can also be used to increase the resistance of foils from other polymers against the permeation of organic solvents and other toxic liquids. The scanning electron microscopy (SEM) microstructure of SiO*_x_* nanocoatings prepared by thermal deposition from SiO in vacuum by the Plasma Assisted Physical Vapour Deposition (PA-PVD) method or vacuum deposition of hexamethyldisiloxane (HMDSO) by the Plasma-enhanced chemical vapour deposition (PECVD) method have been studied. The microstructure and behavior of samples when exposed to a liquid test substance in relation to the barrier properties is described.

## 1. Introduction

**Figure 1:**
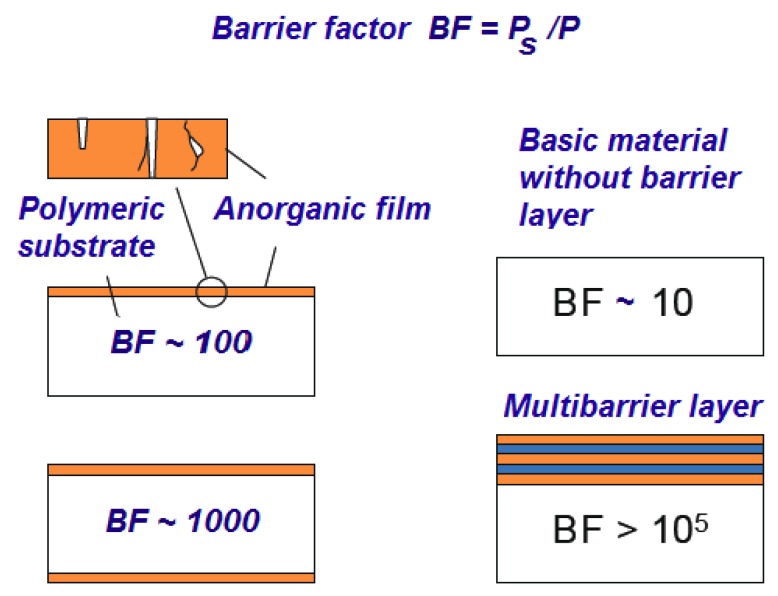
Influence of the barrier nanolayers arrangement on the protective properties of polymeric barrier materials.

Multilayer structures generally increase the protective properties of barrier materials [1,2,3,4]. The effect of a combination of the location of the barrier layers on the Protection Factor (BF) is shown in Figure 1.

Within the production of packaging materials for sensitive commodities and in chemical protection, this principle has been used for many years [5,6]. Multilayer barrier materials usually have better performance properties than the individual components from which they are made [7,8]. They are mostly cheaper and better processed [9,10,11]. This principle is also used by Aluminium Company of Canada (ALCAN) company in the production of SiO*_x_*-based CERAMIS^®^ [12] packaging materials, which form a barrier layer between polymer foils (usually polyethylene terephthalate (PET) and polypropylene (PP)) during their lamination [13,14,15,16]. The principle of this technology of reactivating steaming the SiO*_x_* barrier layer from the SiO substrate in vacuum is illustrated in Figure S1.

In the Aluminium Company of Canada (ALCAN) brochure, this packaging material states that it creates an excellent barrier against gaseous and liquid harmful substances, independent of temperature and humidity. It is transparent for microwaves, suitable for heat sterilization and pasteurization, printable by common printing processes and it allows detection of metals. Due to the patented SiO*_x_* barrier coating technology used by ALCAN was not available, one of the obtained samples was evaluated for protective properties against sulphur mustard (HD) and 1,6-dichlorohexane (DCH). For a CERAMIS^®^ CO 20 sample weighing 18.2 g/m^2^ (PET 12 μm, SiO*_x_* 60 nm, PP 20 μm), the protective properties against the permeation of HD and DCH have been found to be greater than 70 h.

Although this material exhibits high resistance to the permeation of selected toxic compounds, it has one major disadvantage from the point of view of its possible utility in anti-gas protection. Its considerable stiffness and imprecision does not allow it to be used for the construction of protective clothing. It was therefore the effort to apply the SiO*_x_* barrier layer to more suitable polymeric matrices.

Vacuum plasma deposition technology of barrier layers within normal temperatures is particularly suitable for the preparation of advanced surfaces with specific properties on flexible polymer and textile substrates [17,18,19,20]. A synoptical example of this advanced technology is the advanced novoFlex^®^600 equipment for coating polymeric materials in the footage developed by the Fraunhofer Institute for Organic Electronics, Electron Beam and Plasma Technology FEP (Dresden, Germany).

Another way to prepare SiO*_x_* barrier nanolayers on polymeric or textile materials is a method of plasma deposition from a mixture of siloxane substrates and oxygen. As the suitable substrates hexamethyldisiloxane (HMDSO) and tetraethylorthosilicate (TEOS), which most closely approximate to the structure of SiO*_x_* are considered [21].

The barrier layers could be prepared as flexible based on polymeric materials. One of the materials is Parylene. It is a commercial name for the group of poly-*p*-xylylene polymers. Dimmer ofpoly-*p*-xylylene is thermally evaporated (sublimated) from a powder within a low pressure of tens of Pascals. Steams of this dimmer joint a pyrolitic chamber. Within the temperature of approximately 680 °C comes to the split of the dimmer on monomer units. These molecules are reactive and devaporate on the surface of coated materials (in the same way as on walls of the reactive chamber). They polymerize in radical mechanism and form a linear polymeric polymer that is deposited as a very thin barrier layer. This layer is very homogenous and inert. The deposition of parylene was first observed by Szwarc in 1947, and Gorham later described a more efficient, general synthetic way to prepare poly-*p*-xylylene through the vacuum vapour-phase pyrolysis of paracyclophane at has been used till nowadays [22,23]. This polymerisation is self-initiated and non-terminated, requires no solvent or catalyst, produces no by-products except for the unreacted precursor. The polymer thin film is deposited spontaneously at the room temperature thus no thermal stress is induced in a coated object. Recently, parylene is one of the most well-known chemical vapour deposited (CVD) polymers and number of its applications has grown dramatically over the years.

The excellent resistance of polymeric packaging materials with the SiO*_x_* barrier layer against the permeation of oxygen and humidity has conducted to the conception that the plasma deposition of SiO*_x_*nanolayer and/or parylene film on the suitable polymeric matrix could be used for preparation of even constructive materials for protective garments resistant against toxic liquids and gases. This phenomenon has become a subject of our research. The aim of the study was to test these structures on the various polymers be used to increase the resistance against the permeation of organic solvents and other toxic liquids. The swelling of the polymeric substrate and material of nanostructured films is the main problem of barrier films functionality because of some destructions.

## 2. Materials and Methods

### 2.1. Source Materials, Substrates and Polymeric Matrices

SiO powder provided by Merck KGaA (Darmstadt, Germany) was used as source material for Plasma Assisted Physical Vapour Deposition (PA-PVD) process, hexamethyldi-siloxane (HMDSO) CAS 107-46-0 (Sigma-Aldrich, Prague, Czech Republic) was utilized as source materials for PE-CVD method. For PVD process of organics film the 1,3,5-triazine-2,4,6-triamine (melamine), CAS 108-78-1 (Sigma-Aldrich, Prague, Czech Republic) was used. Dimer of chlorinated *p*-xylylene (parylene C) (KunShanZhangShengNaNo Tech Company, Kunshan, China), CAS 28804-46-8 was utilized as source material for CVD method. Bis(2-chloroethyl) sulphide (sulphur mustard, HD) CAS 505-60-2 (Military Technical Institute of Protection, Brno, Czech Republic) and 1,6-Dichlorohexane CAS 218-491-7 (Sigma-Aldrich, Prague, Czech Republic) were used as test chemicals for verification of the quality of new developed barrier materials with nanolayers. Polyvinylidenchlorid (PVDC), CAS 9002-85-1 foil of 20 µm was used for lamination of PEVA foil. Thin films were prepared on polymeric foils from polyethylenevinyl acetate (PEVA), CAS 24937-78-8, foil of 200 µm thickness, polypropylene (PP) foil of 30 um thickness and polyethylentereftalate (PET) foil of 8 µm thickness. Si wafer WFP 6005 provided by ON Semiconductor (ON Semiconductor Czech Republic, Rožnov pod Radhoštěm, Czech Republic) was used as substrate for thin film characterization by energy-dispersive X-ray spectroscopy—EDX (ON Semiconductor Czech Republic, Ltd., Rožnov pod Radhoštěm, Czech Republic). EDX is a part of Scanning Electron Microscope ZEISS EVO LS 10 (Oberkochen, Germany). This spectroscopy provides elemental information about the composition of the structure of the surface of a sample. Performed in conjunction with SEM. Elements with atomic numbers down to carbon can be viewed with Energy Dispersive Spectroscopy (EDS).

### 2.2. Apparatuses for the Samples Preparation

The equipment for thermal lamination of plastic films without the use of binders was used as method for special multilayer structure fabrication. The original vacuum apparatus (Faculty of Chemistry, Brno University of Technology) for the preparation of barrier nanocoatings base on SiO*_x_* and melamine on a polymeric matrix by the PVD, PA-PVD and PECVD method was used. The construction is evident from Figure S2.

Plasma surface activation was carried out before each first layer preparation under pressure of 10 Pa and oxygen flow of 10 sccm. The plasma discharge RF power of 13.56 MHz was set to 50 W for 10 min.

PVD process of SiO and Melamine based layers was carried out in following steps. The vacuum system was evacuated to base pressure of 1 × 10^−5^ Pa, then process started at pressure 1 × 10^−4^ Pa of source material vapour. Sample thickness varied from 50 to 150 nm controlled by deposition time.

PA-PVD process of SiO*_x_* based films preparation was carried out under process pressure of 3 × 10^−2^ Pa and oxygen mass flow of 5 sccm. The RF power of 13.56 MHz plasma discharge was 200 W. The sample thickness varied from 50nm to 150 nm and was controlled by adjusting the deposition time.

PE-CVD process of SiO*_x_* based films deposition from HMDSO vapour was used under following conditions. The process pressure of 8–10 Pa was controlled by pump speed and monomer HMDSO flow, argon mass flow was kept as constant of 2 sccm and oxygen mass flow of 5 sccm. RF power of 13.56 MHz was used in range of 10–100 W, typical Self Bias Voltage was around 100 V. Sample thickness varied from 50–150 nm and was controlled by tuning the deposition time.

For the CVD process of parylene film preparation the original vacuum system developed on Brno University of Technology (Brno, Czech Republic) was used. It works on the principle of Gorham method described above. The conditions used for the process were as follows: pressure of monomer vapour was controlled to 8–10 Pa by temperature of dimer evaporator, the temperature of pyrolytic chamber was kept to 680 °C. The sample thickness was controlled by initial weight of dimer load.

### 2.3. Measuring Instruments

The high-resolution JEOL-6700F scanning electron microscope (JEOL, Tokyo, Japan) was used for the evaluation of nanocoatings surface microstructure at Institute of Scientific Instruments of the Czech Academy of Sciences, Brno, Czech Republic. This microscope is suitable for observing fine structures, such as multi-layer coatings and nanoparticles produced by nanotechnologies. Thanks to very slow electrons, it is also suitable for observing non-conducting samples.

The original SorpTest device was utilized for the study and quantitative testing of the barrier materials resistance against the permeation of volatile toxic substances, including chemical warfare agents [24]. The SorpTest devise is a semiautomatic measuring system using an innovated PVDF permeation cell enabling continual monitoring dynamic and static permeation rate of gases of volatile toxic compound through barrier materials with the employment of the reversible quartz crystal microbalance (QCM) sensor with the polymeric detection layer. With the help of this device it is possible to determine the period during which the limit of the toxic substance penetrates through the barrier material. The rate of permeation of vapours of the tested chemical through the barrier material within its contamination with the liquid phase is monitored. Schema of the SorpTest system is presented Figure S3.

The original equipment developed at the Faculty of Chemistry of the Brno University of Technology was used to determine oxygen transmission rate (OTR) under static conditions. The principle of measurement is similar to principle given in American Society for Testing Material (ASTM) D3985-17. OTR is the steady state rate at which oxygen gas permeates through a film at specified conditions of temperature and relative humidity. Values are expressed in cc/m^2^/24 h units.

## 3. Results and Discussion

### 3.1. Protective Properties

As a carrier material for barrier layers, PEVA has been used as a representative of an easily swellable polymer and PP as a representative of a low swellable polymer. Barrier layers of melamine, parylene, PVDC and SiO*_x_* have been applied to them occasionally combined with each other [25]. The prepared samples have been evaluated from the point of view of protective properties, microstructure and behaviour when exposed to the liquid phase of the test chemical. The PET foil is not suitable as the carrier material; otherwise testing the protective properties would be unbearably long.

**Figure 2:**
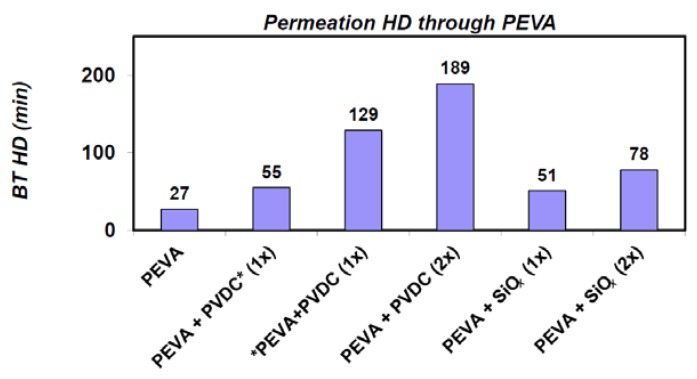
Breakthrough times of modifiedpolyethylenevinyl acetate (PEVA) foil determinate by the MikroTest method.

Combinations of PEVA material with layers of parylene (by CVD), PVDC (by lamination) and SiO*_x_* (PE-CVD) have been prepared and evaluated. Due to the low protection properties of PEVA foil itself against liquid toxic substances, in our case, sulphur mustard (HD) and 1,6-dichlorohexane (DCH), the protective properties of these materials have been verified within one and both-sides coating with a thin layer of PVDC and SiO*_x_*. For one-sided coating, the PVDC has a breakthrough time value (BT_Y_) of 55 min and 129 min from the site of PEVA. This difference has been caused due to the presence of the directional effect of permeation caused by the different swell ability of both materials. The PEVA foil itself had the RD_Y_ only 27 min. For both-sided PVDC lamination, BT_Y_ was found to be 188 min on one side and the second side. For DCH, these values are slightly lower and approximately equal to 51 min on PEVA side and 78 min on both sides covering from both sides (Figure 2).

**Figure 3:**
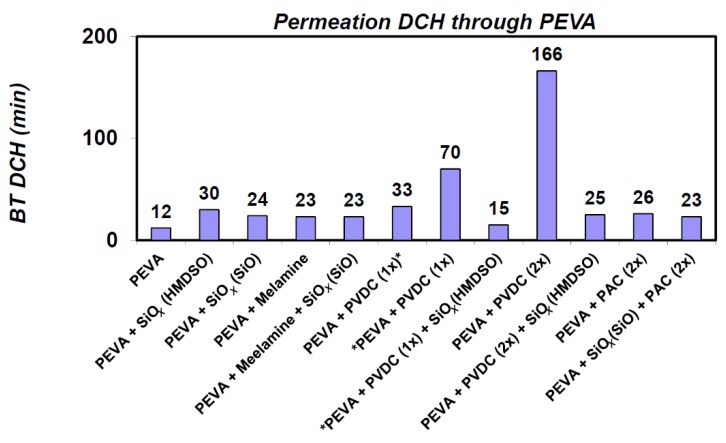
The resistance of the PEVA foil modified with different barrier layers against 1,6-dichlorohexane (DCH).

Further combinations of PEVA foil with barrier layers of SiO*_x_* (by PE-CVD or PA-PVD), melamine (by PVD) and PVDC (by lamination) have been prepared [26,27]. These have been subsequently evaluated for resistance against the permeation of DCH. Interestingly, the resulting BT values for DCH in most combinations have been approximately the same and ranged from 23 to 33 min. The exception was only a combination with PVDC, with a single-sided coverage of BT 70 min from the PEVA side and 166 min with double-sided coverage. These results indicate that the exposure to DCH results in the rapid destruction of most barrier layers as a result of swelling of the carrier polymer so that the resulting protective properties of the starting material do not increase too much (Figure 3). The change in microstructure of the barrier layers following the DCH exposure described below also corresponds to these considerations.

**Figure 4:**
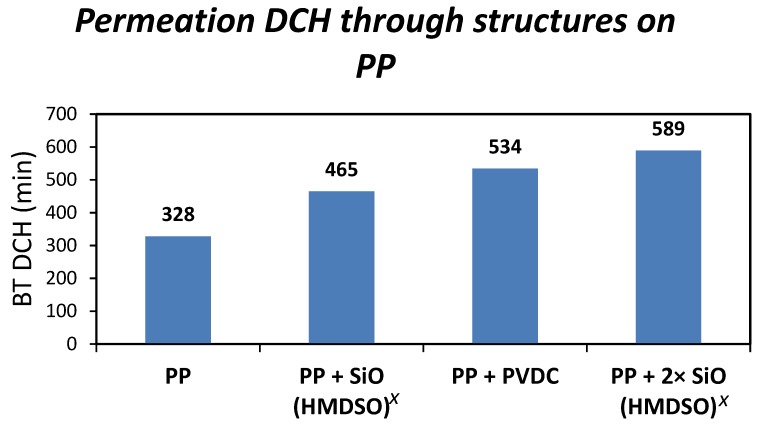
Resistance of the PP foil modified with different barrier layers against DCH permeation.

In order to remove the impact of the swelling on the protective properties of the studied barrier layers, polypropylene has been used as the carrier polymer. The results shown in Figure 4 confirm previous considerations of the negative effect of the swelling of the carrier polymer on the protective properties of the applied barrier nanolayers.

**Figure 5:**
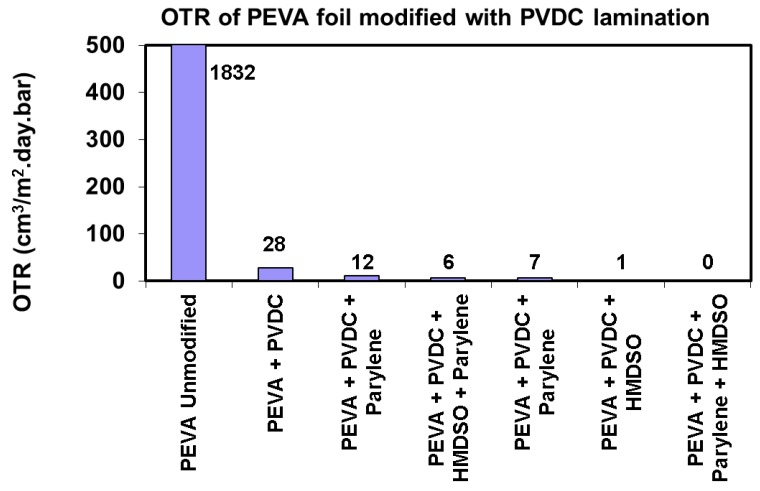
Oxygen permeation values detected for PEVA foil with barrier layers of PVDC, plasma polymerized HMDSO and parylene.

A similar situation occurs when assessing the protective properties with the help of oxygen permeation (OTR), which also does not come to swelling of the carrier polymer (Figure 5).

### 3.2. Microstructure

The analysis is mainly focused on microstructure assessment of inorganic nanocoatings SiO*_x_*, which, when exposed to liquid test chemicals, unlike organic nanocoatings, resulted in significant destruction due to swelling of the polymer matrix. From a large number of SEM micrographs have been selected primarily those that were typical for the studied material and regularly repeated on different samples. Within large magnification, most researched SiO*_x_* layers were found to have characteristic granular substructures of tens of nanometres dimensions, which are the basic building blocks of these nanocoatings.

It is necessary to realize that within the plasma deposition of nanostructured SiO*_x_* barrier layers is a key parameter their elasticity and ductility, which usually is not more than 3% [27,28]. With gradual deformation of the substrate, the effect of ductility on protective properties can also be evaluated by OTR measurements, as there is no swelling of the carrier polymer which causes cracking and thus deterioration of the protective properties. When exposed to a liquid test chemical, the evaluation of the quality of the barrier layers is strongly dependent on the properties of the carrier substrate [29,30]. If a polymer resistant to swelling is used, the results of the protective properties evaluation are similar to those of the OTR. When using an easily swellable polymer, the cracks and defects in the barrier layer polymer are rapidly contaminated by the liquid test chemical and the subsequent swelling causes further destruction of the barrier layer and thereby deterioration of the protective properties.

However, the thickness of the barrier layer is also a crucial parameter. Basically, all publications dealing with the effect of the SiO*_x_* nanocoating thickness on barrier properties indicate an optimum where OTR values are best. The principle of thickness dependence on OTR is explained by the fact that in the nucleation area the discrete centres are first formed on the polymer substrate. The best OTR values are then achieved when they are interconnected. However, if a uniform nanolayer is rapidly formed in another region, its thickness is preferably increased and the growth of discontinuous centres is slowed down. However, a faster increase of the thickness of the barrier nanolayer at these sites above the optimum value, however, results in microcracks resulting from internal stress in the layer material which getting the OTR value worse.

**Figure 6:**
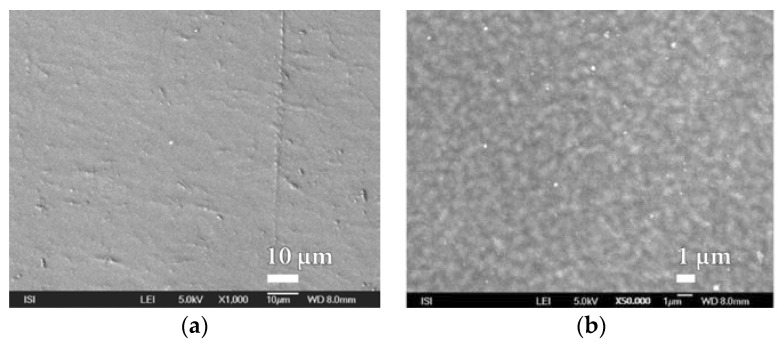
Scanning electron microscopy (SEM) micrographs of the surface of the nanolayer of SiO*_x_* prepared by HMDSO method on the silicon substrate at various magnifications before exposure to DCH (**a**) 1000× and (**b**) 50,000×.

As a basic comparison layer, a nanostructured SiO*_x_* layer on the silicon substrate has been prepared by PECVD by plasma deposition. It is apparent from Figure 6 and Figure S6 that the prepared nanocoating is homogeneous with a uniform microstructure that does not change even after prolonged exposure to DCH.

**Figure 7:**
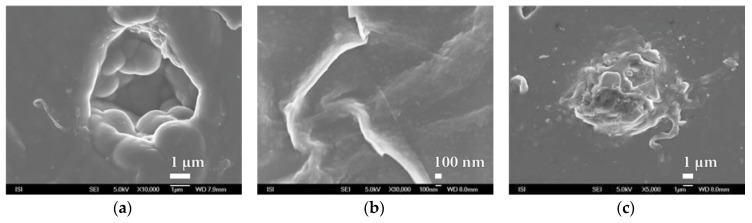
The exhibitions of different inhomogeneity on the surface of the used polymer foil PEVA at various magnifications of (**a**) 10,000×; (**b**) 30,000×;(**c**) 5000×.

When assessing the quality of the SiO*_x_* nanocoatings, the surface quality of the carrier polymer foil should be taken into account in order to determine if found defects do not copy the underlying material. Figure 7 and Figure S7 shows the typical non-homogeneity of the surface of the PEVA foil to be taken into account when assessing the microstructure of SiO*_x_* nanocoatings.

**Figure 8:**
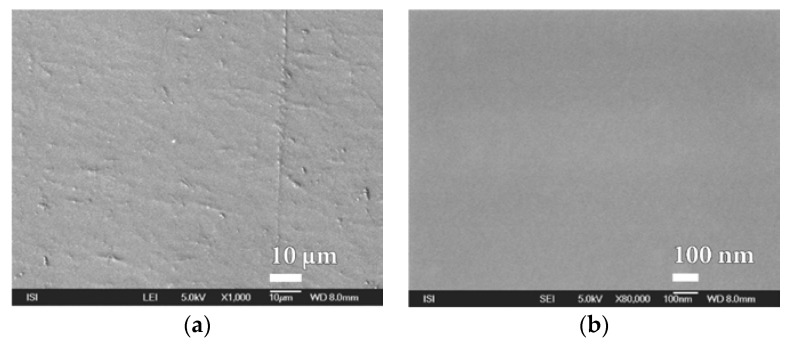
SEM micrographs of PVDC foil surface laminated at 110 °C to PEVA foil at magnification of (**a**) 1000× and (**b**) 80,000×.

For this reason, efforts were made to optimize plasma deposition conditions in order to improve the surface homogeneity of the polymer film used. One from the options was to modify the polymer matrix for less swelling. After verifying a number of PEVA surface treatment options, it was finally chosen to laminate it (at 110 °C without the binder) with a thin PVDC foil that showed good adhesion to the material. Another option was to replace the PEVA foil with a less-swelling PP film with a better surface. The use of PET foil that would be most suitable for plasma barrier coating was problematic because we could not measure too high BT values changes in protective properties in real time after applying different barrier layers. Polymeric matrices thus prepared were subjected to SiO*_x_* barrier nanocoatings with plasma deposition. Prepared samples have been evaluated not only from the point of permeation resistance and for microstructure of barrier nanocoatings surface before and after exposure of the DCH test chemical with high resolution SEM. It is evident from Figure 8 and Figure S8 that the quality of the PVDC surface is significantly better than the original PEVA foil. It has been shown that this material is not porous even at high magnification (80,000×) and therefore it can be advantageously used for the deposition of other barrier layers based on melamine, parylene and mainly SiO*_x_* plasma technology.

**Figure 9:**
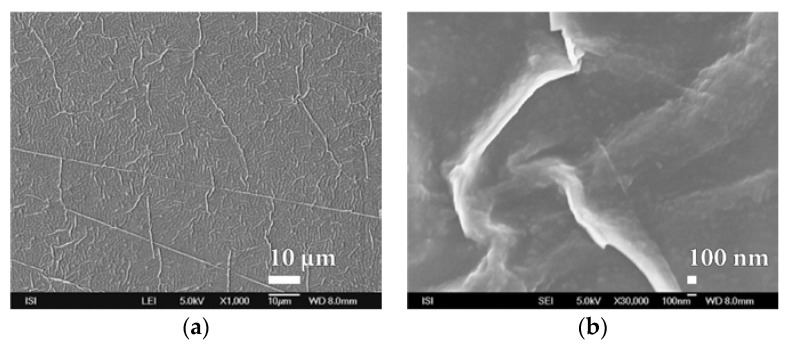
SEM micrographs of PVDC foil surface laminated to PEVA foil after short exposure to oxygen plasma treatment at magnification of (**a**) 1000× (**b**) 30,000×.

However, it is interesting to note that even with short-term oxygen plasma treatment, the surface of the PVDC foil is significantly wrinkled, while its homogeneity remains unchanged (Figure 9 and Figure S9).

**Figure 10:**
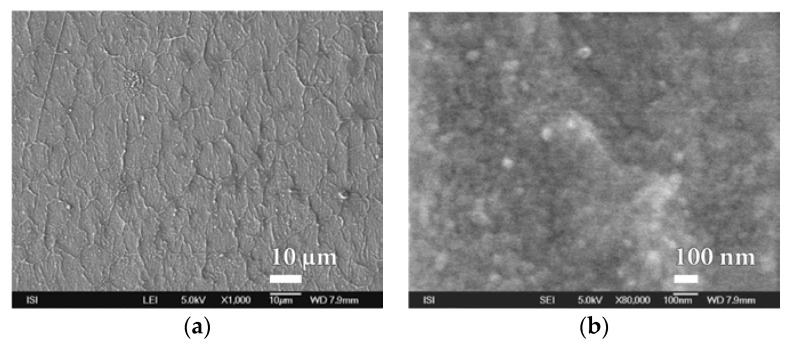
SEM micrographs of the surface SiO*_x_* barrier nanocoating prepared by plasma deposition with the PVD method on PVDC foil laminated on the PEVA foil, at magnification of (**a**) 1000× and (**b**) 80,000×.

A homogeneous layer of SiO*_x_* with a well-developed microstructure which is probably very thin is formed with plasma deposition by PVD method on the surface of the PVDC foil (Figure 10 and Figure S10).

**Figure 11:**
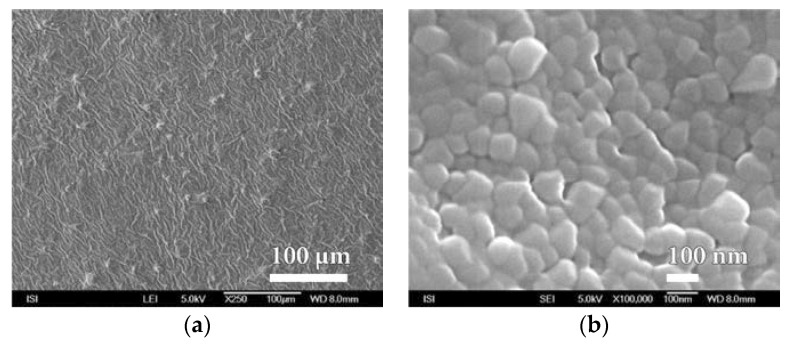
SEM micrographs of melamine nanocoating surface on PEVA SiO*_x_* foil prepared by PVD plasma deposition method at magnification of (**a**) 250× and (**b**) 100,000×.

**Figure 12:**
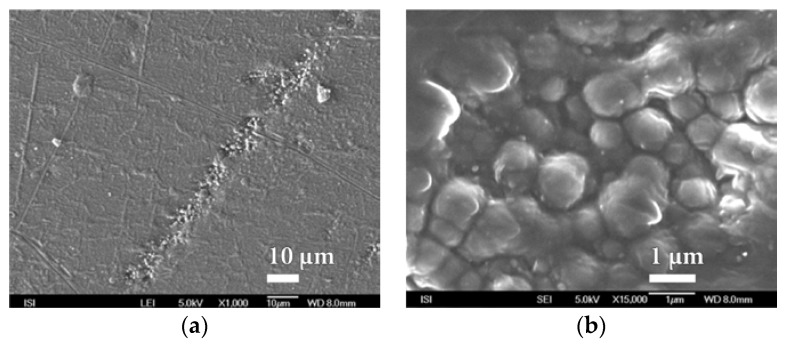
SEM micrographs of parylene nanocoating surfaces on PEVA SiO*_x_* foil prepared by PVD plasma deposition method at magnification of (**a**) 1000× and (**b**) 15,000×.

Similarly, within plasma deposition with the PVD method, at this time on the surface of PEVA foil, behaves even melamine and parylene C. The characteristic microstructure of the surface of these barrier nanocoatings is shown in Figure 11 and Figure 12. In both cases a compact and fairly homogeneous barrier nanocoating with a well-developed microstructure copying the PEVA foil surface is formed. A comparison of the microstructures in Figure 10, Figure 11 and Figure 12 and Figure S10–S12 show that melamine produces a coarser microstructure than SiO*_x_* but thicker than parylene C. Further SiO*_x_* nanocoatings on PEVA film have been prepared by plasma deposition with the HMDSO method. Within this method of preparation, it is possible, in accordance with conditions during exposure, to form a SiO*_x_* nanocoating of the different character than within plasma deposition by PVD method.

**Figure 13:**
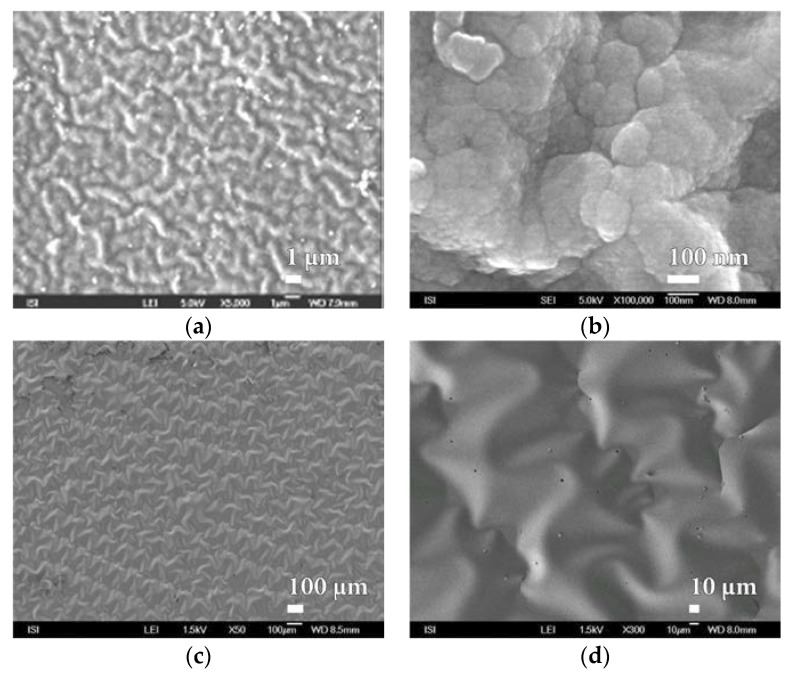
SEM micrographs of SiO*_x_* barrier nanocoating surface prepared by the plasma deposition by HMDSO method on PEVA foil at magnification of (**a**) 5000× and (**b**) 100,000× and on PEVA foil stained with aluminium at magnification of (**c**) 50× and (**d**) 300×.

The different appearance of SiO*_x_* nanocoatings prepared under various experimental conditions, documented in Figure 13 and Figure S13, is probably related to the nanocoating thickness and properties of the carrier substrate.

**Figure 14:**
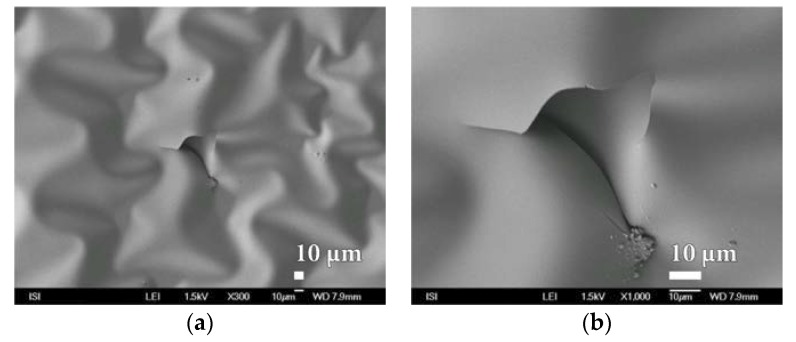
SEM micrographs of surface SiO*_x_* barrier nanocoating prepared by plasma deposition by HMDSO on the silicon wafer at magnification of (**a**) 300× and (**b**) 1000×.

This assumption confirms the deposition of SiO*_x_* by the HMDSO method on the silicon wafer surface, documented in Figure 14 and Figure S14.

**Figure 15:**
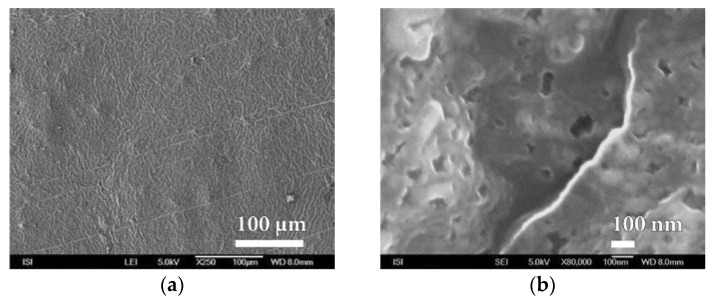
SEM micrographs of surface SiO*_x_* barrier nanocoating prepared by plasma deposition by HMDSO method on the Viton fluorelastomer at magnification of (**a**) 250× and (**b**) 80,000×.

The SiO*_x_* nanocoating has been deposited on the surface of the Viton elastomer by the HMDSO method for comparison, documented in Figure 15 and Figure S15. Interestingly, in this case a homogeneous barrier layer has been formed, however with a porous microstructure.

**Figure 16:**
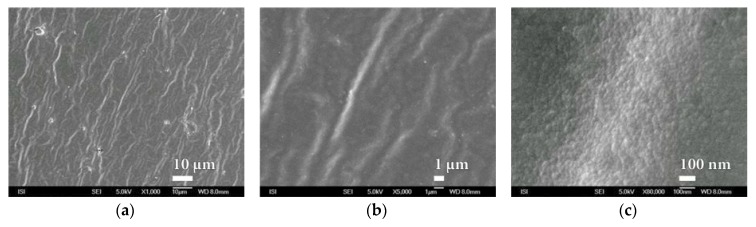
SEM micrographs of SiO*_x_* barrier nanocoating surface prepared by plasma deposition by HMDSO method on the PP foil at magnification of (**a**) 1000×, (**b**) 5000× and (**c**) 80,000×.

The nanocoating SiO*_x_* has been deposited on the surface of both PP and PET foils by the HMDSO method. The formed nanocoating has been both nonporous and homogeneous with a well-developed microstructure copying the surface of the PP foil, as shown in Figure 16.

If samples of polymeric materials with a SiO*_x_* barrier layer are exposed to liquid DCH, an easily swellable PEVA polymer matrix will dramatically change the appearance of this nanocoating. Liquid DCH permeating through micro cracks or other leaks to the polymeric matrix apparently will not prevent its rapid swelling which causes destruction of the SiO*_x_* nanopowder [30,31,32]. This effect will not appear at all or only to a small extent in the scope of PP foils or PET foils. It is conformable with the discussion above related to the protective properties of these materials. The destruction of the SiO*_x_* barrier layer due to the polymer matrix embossment can be demonstrated by many of the examples shown in Figure S4. However, visual changes due to the polymer matrix swelling from visual changes due to mechanical damage to the SiO*_x_* barrier layer, as documented in Figure S5, are required.

**Figure 17:**
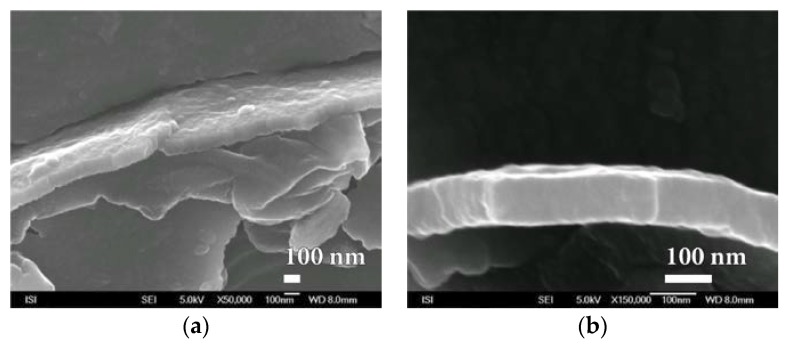
SEM photographs of SiO*_x_* barrier nanocoating fracture prepared by plasma deposition by HMDSO method on PEVA foil at magnification of (**a**) 50,000× and (**b**) 150,000×.

During the study of SiO*_x_* nanocoatings after destruction, sites with such great damage have been found. It was possible to precisely determine the thickness of deposited barrier layer moving around 100 nm (Figure 17 and Figure S16). It even appears that in some cases the SiO*_x_* nanocoating has a multilayer character of approximately 100 nm (Figure 17a).

**Figure 18:**
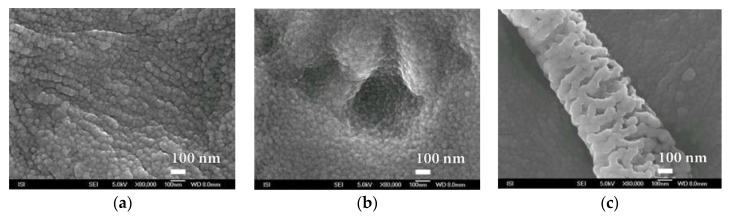
SEM micrographs of SiO*_x_* nanocoating substructure prepared by plasma deposition by HMDSO method on the PEVA foil at magnification of 80,000× of three different areas. (**a**) Typical surface morphology; (**b**) layer defect and (**c**) surface abnormality.

Another interesting finding is the typical spherical substructure characteristic of most studied SiO*_x_* nanocoatings. This substructure, clearly recognizable only with sufficiently large magnification, is also found inside deep material defects and even creates interesting structures on the surface and on the smooth surface of the PEVA foil, as can be seen from Figure 18.

## 4. Conclusions

It has been shown that the plasma deposition in vacuum can form barrier nanopowders from SiO*_x_* and other suitable substrates on the polymer matrix. It is clear from the results that the HMDSO method, which makes it possible to influence the SiO*_x_* nanocoating thickness and quality better, is more appropriate. Within forming practically applicable barrier materials with nanocoating of SiO*_x_* it is necessary to be respect several essential requirements. The nanocoating SiO*_x_* must be deposited primarily on a low swellable polymeric foil and, due to its brittleness, it must immediately be laminated with another foil due to prevention of its mechanical damaged. This is in a harmony with the production process of ALCAN Company within the production of packaging with barrier properties of CERAMIS^®^ technology. The aim of further research in this field should be to improve the fixation of barrier nanocoatings on the polymer matrix and their resistance to mechanical stress. If it is real to prepare reactive coatings or deposition of suitable metallic oxides with catalytic properties it would enable this process to be used for the preparation of nanocoatings and nanomaterials with self-decontaminating or photocatalytic properties during further research.

## Supplementary Materials

The following are available online at http://www.mdpi.com/2079-4991/8/9/679/s1, Figure S1: The principle of CERAMIS^®^ reactive steam technology and examples of SiO*_x_*^®^ coated materials, Figure S2: The scheme of the PE-CVD/PA-PVD apparatus for the preparation of SiO*_x_*nanocoatingsSiO*_x_* with steaming form powder SiO in vacuum, Figure S3: Diagram and real appearance of the SorpTest device for testing the resistance of barrier materials against the permeation of gaseous and liquid toxic substances, Figure S4: Scanning electron microscopy (SEM) micrographs of examples of visual changes of the SiO*_x_* barrier layer caused by polymer matrix swelling with visible different kinds of cracks, Figure S5: Scanning electron microscopy (SEM) micrographs of examples of visual changes of the SiO*_x_* barrier layer caused by mechanical damage, Figure S6: Scanning electron microscopy (SEM) micrographs of the surface of the nanolayer of SiO*_x_* prepared by HMDSO method on the silicon substrate at various magnifications of 20,000× before exposure to DCH, Figure S7: The exhibitions of different inhomogeneity on the surface of the used polymer foil PEVA at various magnification of (a) 30,000×, (b) 15,000×, (c) 80,000×, Figure S8: SEM micrographs of PVDC foil surface laminated at 110 °C to PEVA foil at magnification of 30,000×, Figure S9: SEM micrographs of PVDC foil surface laminated to PEVA foil after short exposure to oxygen plasma treatment at magnification of 5000, Figure S10: SEM micrographs of the surface SiO*_x_* barrier nanocoating prepared by plasma deposition with the PVD method on PVDC foil laminated on the PEVA foil, at magnification of 5000×, Figure S11: SEM micrographs of melamine nanocoating surface on PEVA SiO*_x_* foil prepared by PVD plasma deposition method at magnification of 30,000×, Figure S12: SEM micrographs of parylenenanocoating surface on PEVA SiO*_x_* foil prepared by PVD plasma deposition method at magnification of 5000×, Figure S13: SEM micrographs of SiO*_x_* barrier nanocoating surface prepared by the plasma deposition by HMDSO method on PEVA foil at magnification of (a) 1000× and on PEVA foil stained with aluminium at magnification (b) 1000×, Figure S14: SEM micrographs of surface SiO*_x_* barrier nanocoating prepared by plasma deposition by HMDSO on the silicon wafer at magnification of 1000×, Figure S15: SEM micrographs of surface SiO*_x_* barrier nanocoating prepared by plasma deposition by HMDSO method on the Viton fluorelastomer at magnification of 5000×, Figure S16: SEM photographs of SiO*_x_* barrier nanocoating fracture prepared by plasma deposition by HMDSO method on PEVA foil at magnification of 100,000×.
