# Supplementary material for: Protective Properties of a Microstructure Composed of Barrier Nanostructured Organics and SiOx Layers Deposited on a Polymer Matrix
Source: Nanomaterials (Basel). 2018 Aug 31;8(9):679. doi: 10.3390/nano8090679 (PMC6164386; doi:10.3390/nano8090679)
Supplement: Supplementary file 1 [file nanomaterials-08-00679-s001.zip › nanomaterials-326749-SI.pdf]

Protective Properties of A Microstructure Composed of Barrier Nanostructured Organics and SiO_x Layers Deposited on A Polymer Matrix

Radek Prikryl, Pavel Otrisal, Vladimir Obsel, Lubomír Svorc, Radovan Karkalic and Jan Buk

Supporting Information

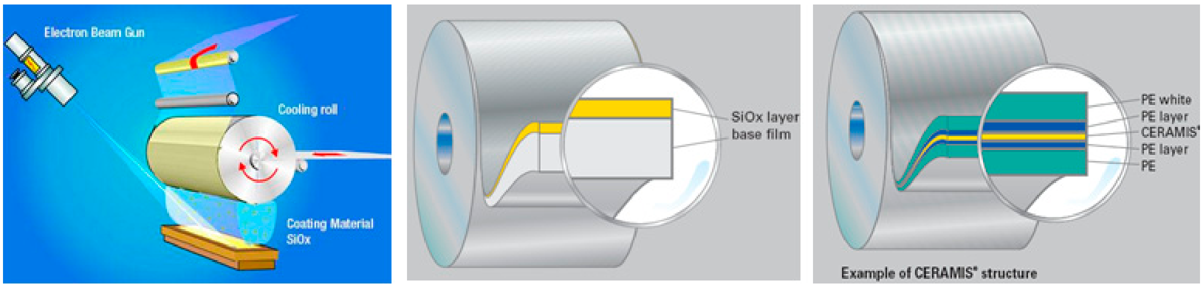

Figure S1. The principle of CERAMIS reactive steam technology and examples of SiO_x® coated materials

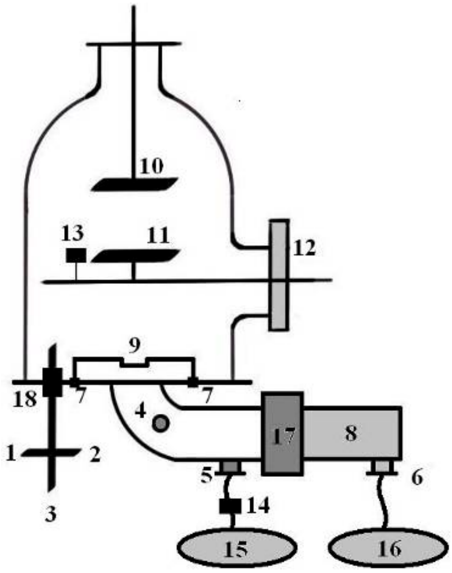

Figure S2. The scheme of the PE-CVD/PA-PVD apparatus for the preparation of SiO_x nanocoatings SiO_x with steaming form powder SiO in vacuum

1-the vapor flow regulator of the monomer (used in the case of PECVD), 2-regulator of oxygen flow, 3-argon flow regulator, 4-flange with attached accessories (Pirani gauge, Pennig gauge, needle metering/venting valve), 5-Flange for oil rotary vacuum pump connection, 6- flange for connecting a large oil rotary pump - the first stage of the pumping system, 7-vacuum electrical current feedthrough, 8-turbo molecular vacuum pump, 9-wolfram or molybdenum boat for the evaporation of solid material, 10-stainless steel live powered electrode, 11- stainless steel rotating electrode, 12- reactor inlet, 13-quarz crystal measurement sensor of film thickness , 14-valve, 15-low vacuum small oil rotary pump, 16- main vacuum pump, 17-controlgate valve, 18-gas feedthrough.

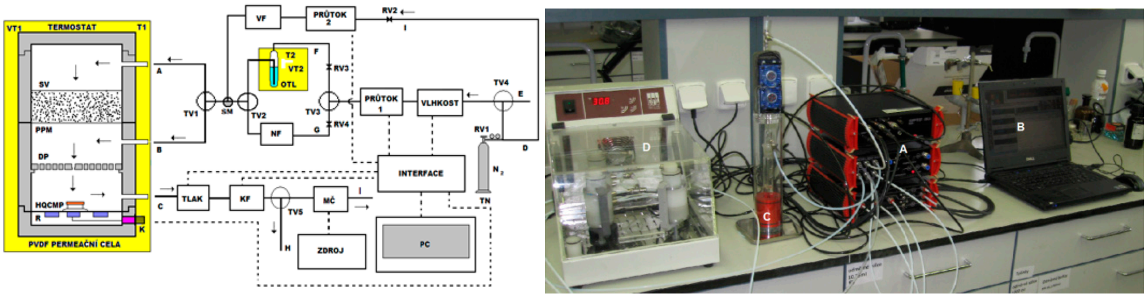

Figure S3. Diagram and real appearance of the SorpTest device for testing the resistance of barrier materials against the permeation of gaseous and liquid toxic substances

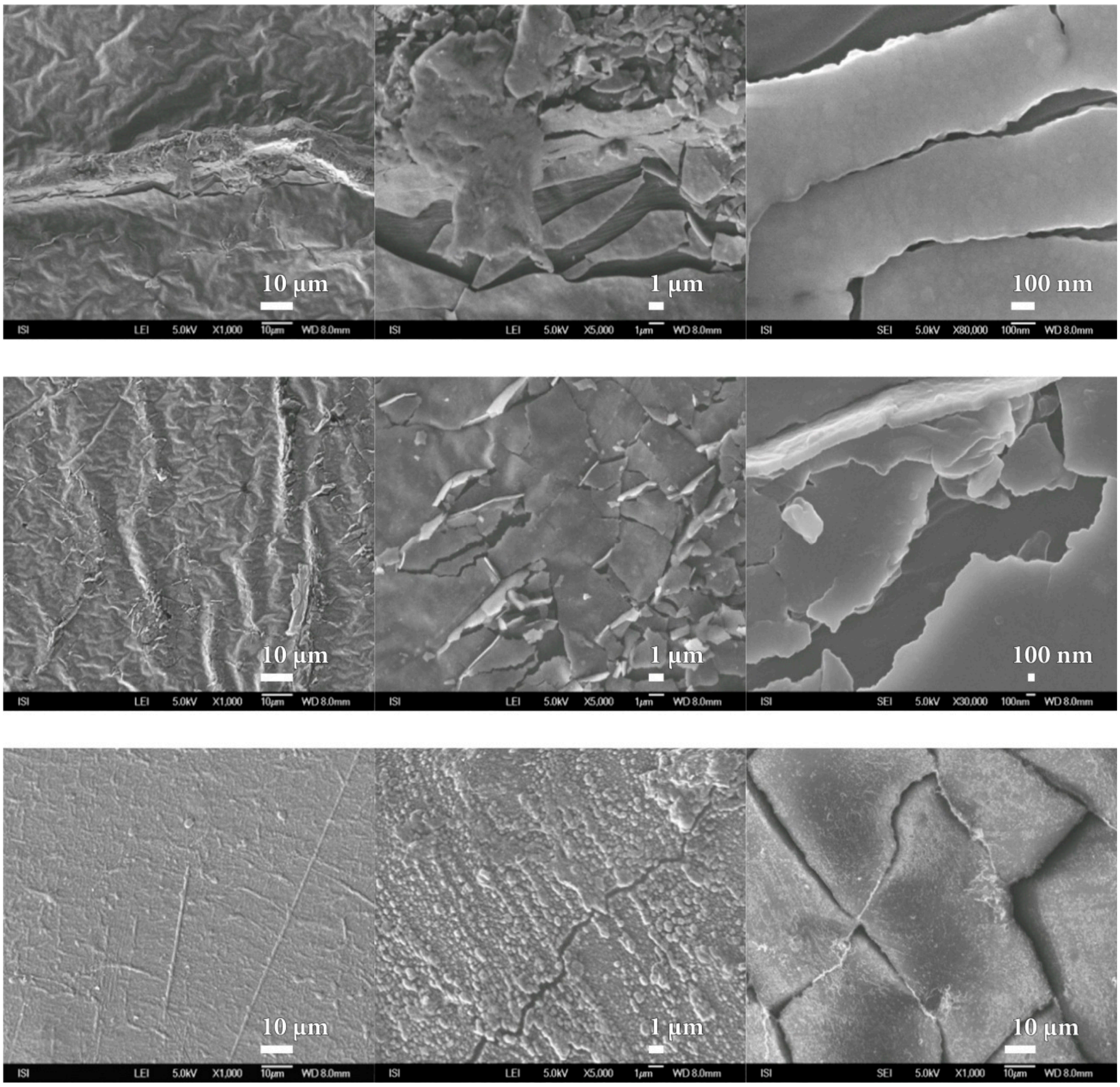

Figure S4. Scanning electron microscopy (SEM) micrographs of examples of visual changes of the SiO_x barrier layer caused by polymer matrix swelling with visible different kinds of cracks

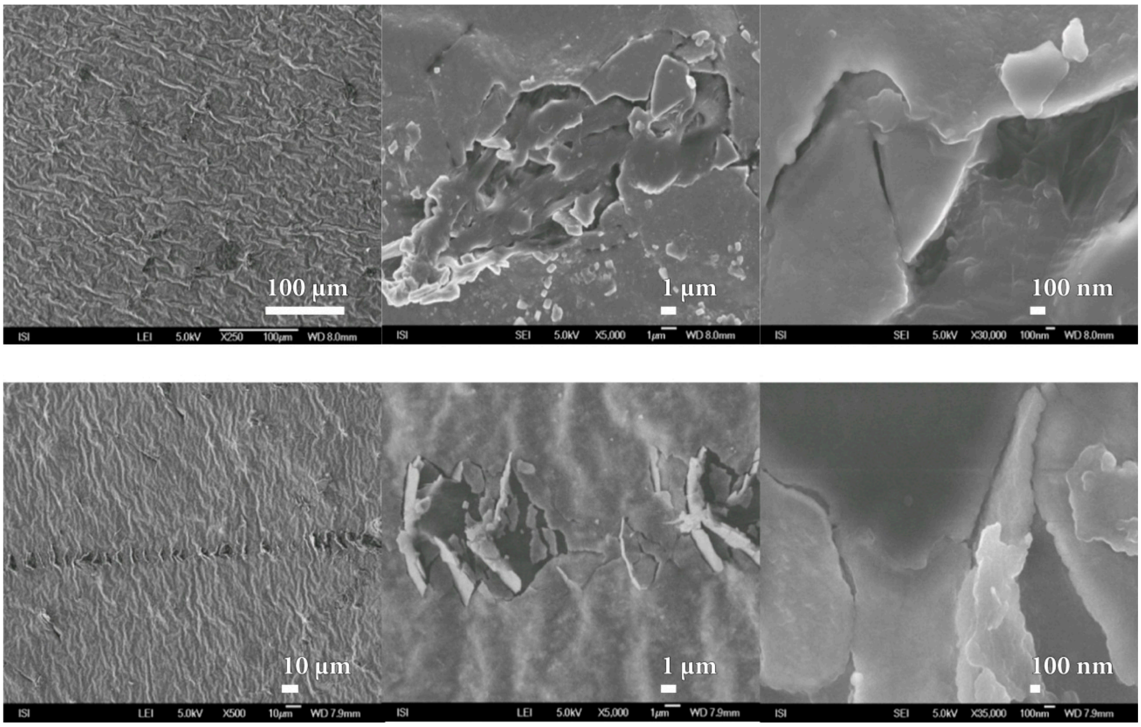

Figure S5. Scanning electron microscopy (SEM) micrographs of examples of visual changes of the SiO_x barrier layer caused by mechanical damage.

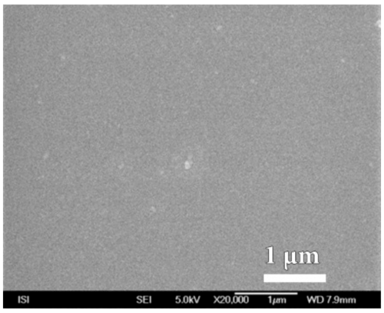

Figure S6. Scanning electron microscopy (SEM) micrographs of the surface of the nanolayer of SiO_x prepared by HMDSO method on the silicon substrate at various magnifications of 20,000× before exposure to DCH

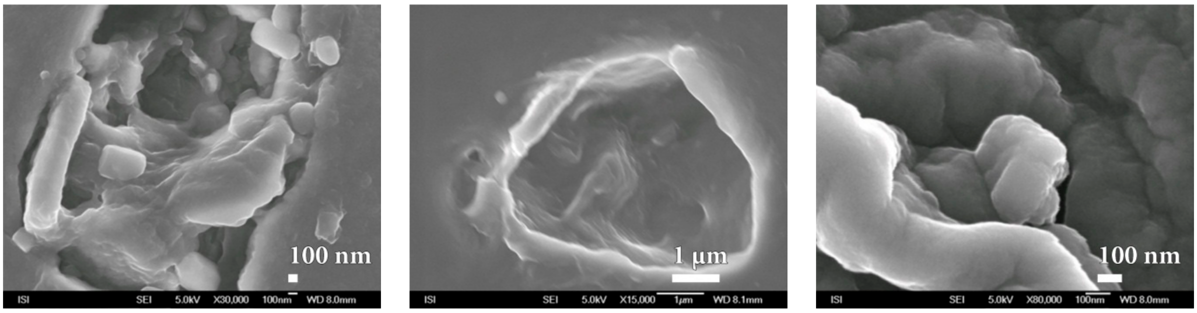

(**a**) (**b**) (**c**)

Figure S7. The exhibitions of different inhomogeneity on the surface of the used polymer foil PEVA at various magnification of (**a**) 30,000×, (**b**) 15,000×, (**c**) 80,000×.

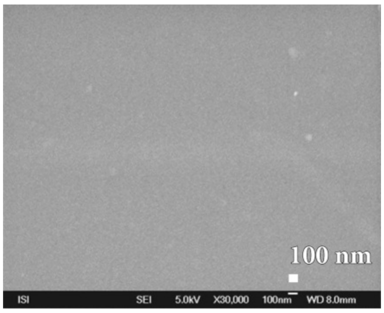

Figure S8. SEM micrographs of PVDC foil surface laminated at 110 °C to PEVA foil at magnification of 30,000×.

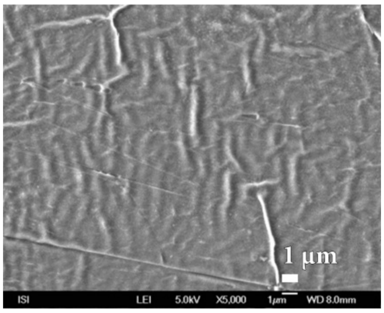

Figure S9. SEM micrographs of PVDC foil surface laminated to PEVA foil after short exposure to oxygen plasma treatment at magnification of 5000×.

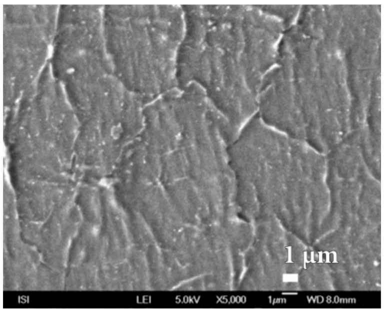

Figure S10. SEM micrographs of the surface SiO_x barrier nanocoating prepared by plasma deposition with the PVD method on PVDC foil laminated on the PEVA foil, at magnification of 5000×.

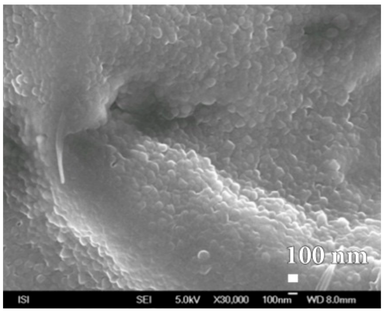

Figure S11. SEM micrographs of melamine nanocoating surface on PEVA SiO_x foil prepared by PVD plasma deposition method at magnification of 30,000×.

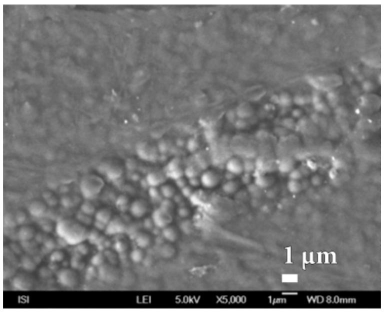

Figure S12. SEM micrographs of parylene nanocoating surface on PEVA SiO_x foil prepared by PVD plasma deposition method at magnification of 5000×.

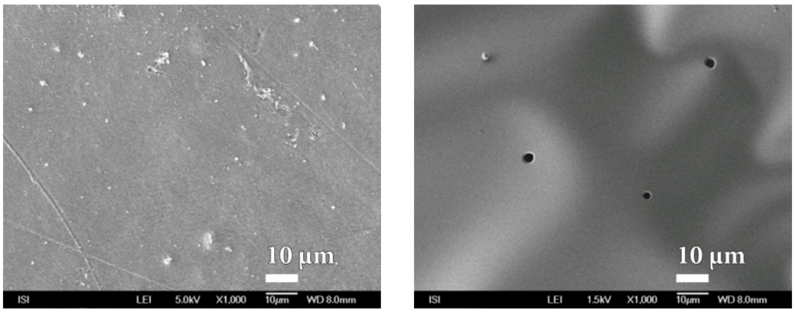

(**a**) (**b**)

Figure 13. SEM micrographs of SiO_x barrier nanocoating surface prepared by the plasma deposition by HMDSO method on PEVA foil at magnification of (**a**) 1000× and on PEVA foil stained with aluminum at magnification (**b**) 1000×.

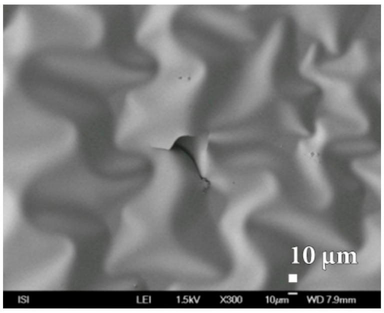

Figure S14. SEM micrographs of surface SiO_x barrier nanocoating prepared by plasma deposition by HMDSO on the silicon wafer at magnification of 300×.

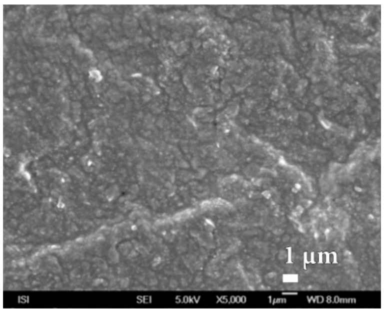

Figure S15. SEM micrographs of surface SiO_x barrier nanocoating prepared by plasma deposition by HMDSO method on the Viton fluorelastomer at magnification of 5000×.

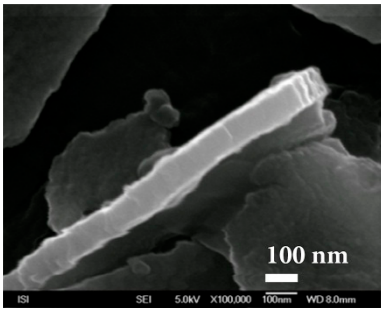

Figure S16. SEM photographs of SiO_x barrier nanocoating fracture prepared by plasma deposition by HMDSO method on PEVA foil at magnification of 100,000×.
